# Evolution of REP diversity: a comparative study

**DOI:** 10.1186/1471-2164-14-385

**Published:** 2013-06-10

**Authors:** Jaroslav Nunvar, Irena Licha, Bohdan Schneider

**Affiliations:** 1 Department of Genetics and Microbiology, Faculty of Science, Charles University, Vinicna 5, 128 44, Prague 2, Czech Republic; 2 Institute of Biotechnology of the ASCR, v. v. i., Videnska 1083, 142 20, Prague 4, Czech Republic

**Keywords:** REP elements, *Stenotrophomonas maltophilia*, *Pseudomonas fluorescens*

## Abstract

**Background:**

Repetitive extragenic palindromic elements (REPs) constitute a group of bacterial genomic repeats known for their high abundance and several roles in host cells´ physiology. We analyzed the phylogenetic distribution of particular REP classes in genomic sequences of sixty-three bacterial strains belonging to the *Pseudomonas fluorescens* species complex and ten strains of *Stenotrophomonas* sp., in order to assess intraspecific REP diversity and to gain insight into long-term REP evolution.

**Results:**

Based on proximity to RAYT (REP-associated tyrosine transposase) genes, twenty-two and thirteen unique REP classes were determined in fluorescent pseudomonads and stenotrophomonads, respectively. In stenotrophomonads, REP elements were typically found in tens or a few hundred copies per genome. REPs of fluorescent pseudomonads were generally more numerous, occurring in hundreds or even over a thousand perfect copies of particular REP class per genome. REP sequences showed highly heterogeneous distribution. The abundances of REP classes roughly followed host strains´ phylogeny, differing markedly among individual clades. High abundances of particular REP classes appeared to depend on the presence of the cognate RAYT gene, and deviations from this state could be attributed to recent or ancient mutations of *rayt*-flanking REPs, or RAYT loss. RAYTs of both studied bacterial groups are monophyletic, and their cognate REPs show species-specific characteristics, suggesting shared evolutionary history of REPs, RAYTs and their hosts.

**Conclusions:**

The results of our large-scale analysis show that REP elements constitute intriguingly dynamic components of genomes of fluorescent pseudomonads and stenotrophomonads, and indicate that REP diversification and proliferation are ongoing processes. High numbers of REPs have apparently been retained during the entire evolutionary time since the establishment of these two bacterial lineages, probably because of their beneficial effect on host long-term fitness. REP elements in these bacteria represent a suitable platform to study the interplay between repeated elements, their mobilizers and host bacterial cells.

## Background

Genomes of many higher eukaryotes are known to teem with repetitive DNA elements. By contrast, bacteria are notorious for their high coding density [1], which leaves significantly less space for expansion of repeats. Repetitive elements identified in bacteria can be generally divided into coding and noncoding ones. The former is typically represented by insertion sequences and transposons, parasitic DNA elements that catalyze their own movement and replication (with help of host cell´s functions) [2]. Noncoding repeats (apart from repeated genes coding for structural RNAs) comprise several distinct types, often connected to various cellular functions. For example, short, overrepresented DNA motifs mark DNA to be taken up by natural transformation in *Haemophilus* and related bacteria [3]. Similarly, Chi sequences, which serve as sites of recombination initiation, are overrepresented in host genomes [4]. Repeated elements are part of sophisticated CRISPR systems, which provide defense against invading mobile elements [5]. Finally, various types of MITEs (miniature inverted repeats transposable elements), which are predicted to be derived from autonomous transposable elements, are implicated in transcription regulation and other processes [6,7].

REP (repetitive extragenic palindrome) elements have now been known for over 30 years [8], originally from *Escherichia coli* and related enterobacteria [9]. They were later identified in other species, belonging predominantly to gammaproteobacteria – *Pseudomonas putida*[10], *Pseudomonas fluorescens*[11,12], *Stenotrophomonas maltophilia*[13], *Xanthomonas campestris* and others [14], each species possessing different types of REP sequences. REPs are typically highly numerous and occur almost exclusively in intergenic regions. The definition of REP elements was recently refined [14] to reflect their common features on sequence level: a 5´-terminal conserved tetranucleotide (GT^A^/_G_G) and downstream complementary (palindromic) region with variable base composition. REP elements are mostly arranged into repeats of higher order. REPINs (REP doublet forming hairpin) are composed of two closely spaced REPs in inverted orientation [15] and were found to represent the predominant REP form in *P*. *fluorescens*[11,15], *P*. *putida*[10] and *S*. *maltophilia*[13]. BIMEs (bacterial interspersed mosaic elements), abundant in *E*. *coli*, consist of tandemly repeated REPIN-like doublets [16]. Importantly, in *E*. *coli*, three significant proteins interact with REPs or BIMEs: integration host factor [17], DNA gyrase [18] and DNA polymerase I [19], indicating the role for these elements in major cellular processes. Furthermore, REPs were shown to modulate transcription and mRNA stability in both *E*. *coli*[20] and *S*. *maltophilia*[13]. REPs inhabit only the core parts of host genomes and are absent from laterally transferred regions [11-13].

A few years ago, we described a protein family associated with REP sequences, RAYTs (REP-associated tyrosine transposases) [14]. Related to transposases of the IS*200*/IS*605* insertion sequence family [21,22], RAYTs carry conserved residues to perform DNA cleavage – the catalytic tyrosine and two metal-coordinating histidines. Since REP elements were found flanking RAYT genes in almost all species where they have been previously recorded, REPs were the likely substrates to be cleaved by RAYTs. The predicted REP-specific nuclease activity of *E*. *coli* RAYT was recently confirmed experimentally [23], and the crystal structure of REP/RAYT complex was solved [24]. The structure helped to elucidate the role of conserved tetranucleotide and palindrome (two defining features of REP elements) in REP recognition by RAYTs.

Owing to rapid expansion of Next-generation DNA sequencing methods, increasing numbers of new genomic sequences are reported each year. These provide great opportunity to conduct comparative analyses. We explored the distribution of REP elements and their associated RAYTs in sequenced genomes of sixty-three fluorescent pseudomonads and ten stenotrophomonads, two groups of omnipresent environmental bacteria with biotechnological and biocontrol applications [12,25]. Our results indicate rapid diversification and proliferation of REPs in both studied groups. Furthermore, RAYTs appear to play a principal role in REP dissemination, as RAYT presence correlates with REP abundance. Our results provide support for the hypothesis that REP/RAYT system is an example of mobile element domestication.

## Results and discussion

### Phylogenetic relationships of studied bacteria

**Figure 1:**
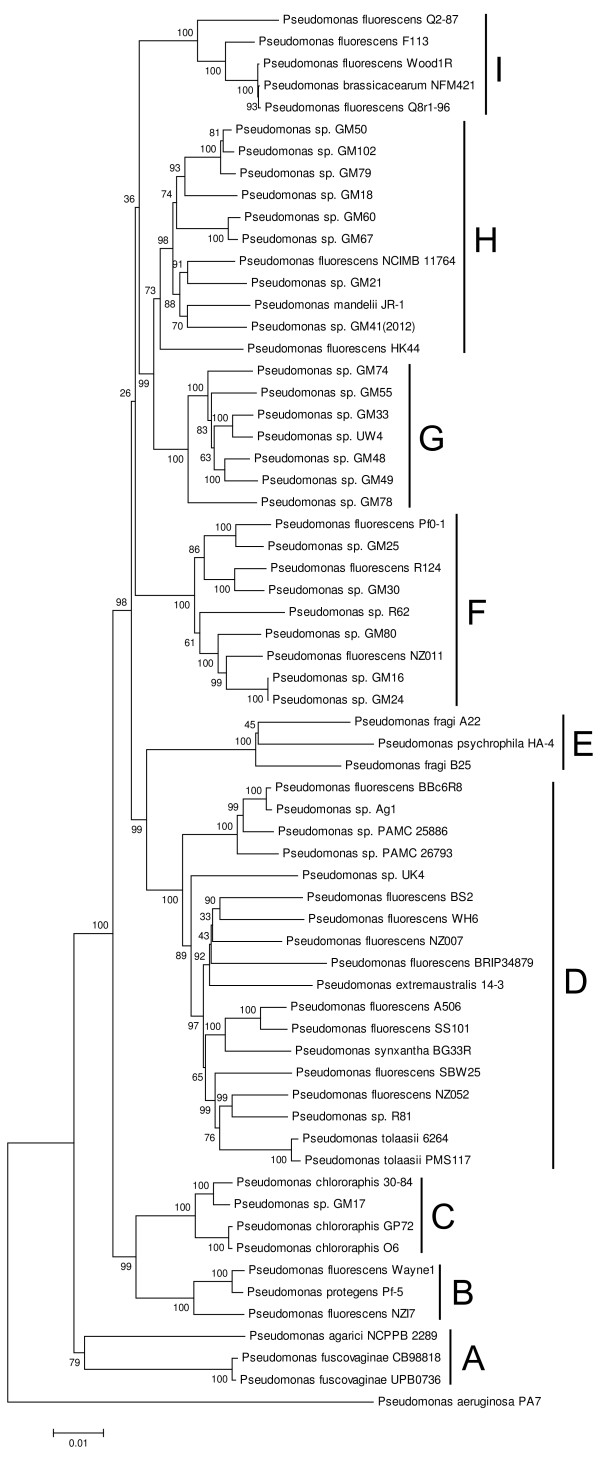
**Neighbor-Joining phylogram of 63 fluorescent pseudomonads.** The tree was constructed from concatenated complete nucleotide sequences of *gyrB*, *rpoB* and *rpoD* genes. Resulting clades are marked with vertical lines to the right of corresponding strains and labeled with letters **A** – **I**.

**Figure 2:**
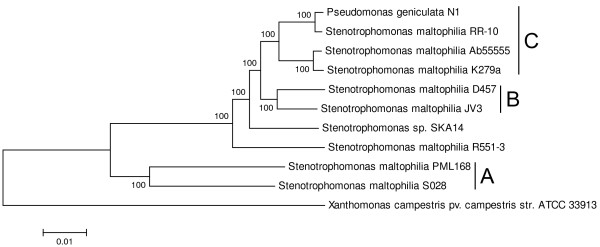
**Neighbor-Joining phylogram of 10 stenotrophomonads.** The tree was constructed from concatenated complete nucleotide sequences of *gyrB*, *rpoB* and *rpoD* genes. Resulting clades are marked with vertical lines to the right of corresponding strains and labeled with letters **A** – **C**.

Our preliminary analysis of available genomes revealed that the greatest intraspecific diversity of REP elements and their associated RAYTs existed in bacteria of the *Pseudomonas fluorescens* complex and in *Stenotrophomonas* sp. (data not shown). Comprehensive mining of bacterial genomic databases recovered 63 genomes affiliated to *Pseudomonas fluorescens* (fluorescent pseudomonads) and 10 genomes affiliated to *Stenotrophomonas maltophilia* (stenotrophomonads). Among fluorescent pseudomonads, species of *P*. *agarici*, *P*. *brassicacearum*, *P*. *chlororaphis*, *P*. *extremaustralis*, *P*. *fragi*, *P*. *fuscovaginae*, *P*. *mandelii*, *P*. *protegens*, *P*. *psychrophila*, *P*. *synxantha* and *P*. *tolaasii*, previously shown to belong to the *P*. *fluorescens* complex [26], were included, as well as numerous *Pseudomonas sp*. isolates, unassigned to any species. For stenotrophomonads, *Pseudomonas geniculata*, synonym for *S*. *maltophilia*[27], was included, as well as *Stenotrophomonas sp*. SKA14. To resolve the evolutionary relationship between the strains, phylogenetic trees were constructed from three housekeeping genes (Figure 1, Figure 2). The phylogram of fluorescent pseudomonads revealed nine well-supported clades (A – I). The phylogram of stenotrophomonads identified three clades (A – C) and two solitary strains. The inter- and intra-clade phylogram resolution was perfect for stenotrophomonads while only partially satisfactory for fluorescent pseudomonads. This difference might be due to the effect of recombination, since *P*. *fluorescens* was shown to be naturally competent for transformation [28], whereas natural competence is unknown in *S*. *maltophilia*.

### Diversity of REP sequences and RAYTs

In the next step, the spectrum of REP elements was determined in genomes of studied strains. For this purpose, we utilized the specific association between RAYT (REP-associated tyrosine transposase) genes and REP elements. This approach (see Methods) yielded twenty-two and thirteen unique classes of REP elements in fluorescent pseudomonads (PF1 – PF22) and stenotrophomonads (SM1 – SM13), respectively (Table 1, Table 2, Additional file 1) For some REP classes, sequence ambiguities were detected when two slightly different REP sequences were associated with the same *rayt* gene. REPs of stenotrophomonads always contain eight or nine perfectly complementary bases, located directly adjacent to the GT^A^/_G_G tetranucleotide. In contrast, in fluorescent pseudomonads REPs, palindromes are flanked by additional two or three nucleotides on both sides and the length of the palindromes is significantly shorter (Table 1, Table 2). The majority of detected REPs occurred as close inverted doublets (REPINs), as reported previously [13,15]. The cognate RAYTs of both bacterial groups are monophyletic (Additional file 2), suggesting that although quite diverse, they have been present in their host genomes for substantial evolutionary time. Intriguingly, several different classes of REP sequences were found to flank orthologous RAYT genes (as judged by their shared chromosomal location - synteny) between related strains in both bacterial sets. These cases were gathered into so called orthogroups. An orthogroup comprises the classes of REP elements associated with synthenic (orthologous) RAYTs. Three orthogroups were detected in stenotrophomonads and four in fluorescent pseudomonads (Table 1, Table 2), of which orthogroup IV is the most numerous and includes nine distinct REP classes (PF8 - PF16).

**Table 1:** Summary information on identified RAYTs and their cognate REP elements in sequenced fluorescent pseudomonads

	**RAYT/REP symbol**	**RAYT accession number**	**Cognate REP sequence**
**Orthogroup I**	**PF1**	YP_002873491 (*P*. *fluorescens* SBW25)	***GTGG***GAGGGGGCTTGCCCCCGAT
	**PF2**	n.a.	***GTGG***GAGGGGGCTTGCTCCCGAT
**Orthogroup II**	**PF3**	n.a.	***GTAG***GAGCyGGCTTGCCrGCGAA
	**PF4**	EJM82571 (*P*. *sp*. GM60)	***GTAG***GAGCCGGCTTGCTGGCGAT
**Orthogroup III**	**PF5**	EJN28792 (*P*. *sp*. GM80)	***GTGG***yGAGGGGATTTATCCCCG
	**PF6**	n.a.	***GTGG***CGAGGGGGCTTGTCCCCCG
	**PF7**	EJM60273 (*P*. *sp*. GM49)	***GTGG***CGAGGGGGCTTGCCCCCG
**Orthogroup IV**	**PF8**	EIK66912 (*P*. *fluorescens* Q8r1-96)	***GTGG***GAGCGAGCTTGCTCGCGAT
	**PF9**	EKA23398 (*P*. *fluorescens* BBc6R8)	***GTGG***GAGCGGGCTTGCTCGCGAA
	**PF10**	EJM47370 (*P*. *sp*. GM33)	***GTGG***GAGCGAGCyTGCTCGCGAA
	**PF11**	n.a.	***GTAG***GAGTGAGCCTGCTCGCGAT
	**PF12**	YP_006323329 ( *P*. *fluorescens* A506)	***GTGG***GAGCTGGCTTGCCTGCGAT
	**PF13**	n.a.	***GTGG***GAGCGGGCTTGCCCGCGAT
	**PF14**	ZP_10622153 (*P*. *sp*. GM78)	***GTGG***GAGCTGGCTTGCCAGCGAT
	**PF15**	EJM57603 (*P*. *sp*. GM41 (2012))	***GTGG***GAGCCAGCCTGCTGGCGAT
	**PF16**	EJM16763 ((*P*. *sp*. GM21)	***GTGG***GAGCTAGCCTGCTAGCGAT
NO^*^	**PF17**	YP_002871781 (*P*. *fluorescens* SBW25)	***GTGG***CGAGGGAGCTTGCTCCCGCT
NO^*^	**PF18**	ZP_10436910 (*P*. *extremaustralis* 14–3)	***GTAG***GAGCGAGCyyGCTCGCGA
NO^*^	**PF19**	YP_004351241 (*P*. *brassicacearum* NFM421)	***GTrG***GAGCAAGGCTTGCCCGCGAT
NO^*^	**PF20**	EJM39110 (*P*. *sp*. GM33)	***GTAG***GAGCTGCCGAAGGCTGCGAT
NO^*^	**PF21**	EIM18788 (*P*. *chlororaphis* O6)	***GTAG***GAGCGAGGCTTGCCCGCGA
NO^*^	**PF22**	YP_002873800 (*P*. *fluorescens* SBW25)	***GTrG***TGAGCGGGCTTGCCCCGCGCT

REP sequences are denoted in 5´ to 3´ orientation as follows: conserved tetranucleotide in bold and italics, complementary (palindromic) nucleotides underlined, variable nucleotides (IUPAC code) in lower case.

^*^ NO – no orthologous RAYT genes flanked by differing REPs were detected.

n. a. – protein not annotated (see Additional file 1 for these sequences).

**Table 2:** Summary information on identified RAYTs and their cognate REP elements in sequenced stenotrophomonads

	**RAYT/REP symbol**	**RAYT accession number**	**Cognate REP sequence**
**Orthogroup I**	**SM1**	YP_001970973 (*S*. *maltophilia* K279a)	G***GTGG***GTGCCGACCGTTGGTCGGCAC
	**SM2**	YP_002708831 (*S*. *sp*. SKA14)	G***GTGG***GTGCCAACCTTGGTTGGCAC
	**SM3**	YP_006183766 (*S*. *maltophilia* D457)	***GTAG***wTGCCAACCTTGGTTGGCA
**Orthogroup II**	**SM4**	YP_002706198 (*S*. *sp*. SKA14)	***GTrG***ATCCACGCCATGCGTGGAT
	**SM5**	n.a.	***GTAG***AGCCACCCCATGGGTGGCT
**Orthogroup III**	**SM6**	n.a.	G***GTAG***AGTCGACTGTTAGTCGACT
	**SM7**	n.a.	***GTAG***mGCCGGGyTCTrCCCGGCk
NO^*^	**SM8**	YP_001972572 (*S*. *maltophilia* K279a)	G***GTAG***TGCCGGCCGCTGGCCGGCA
NO^*^	**SM9**	YP_002030358 (*S*. *maltophilia* R551-3)	T***GTAG***AGCCGAGCCCATGCTCGGCT
NO^*^	**SM10**	YP_002029847 (*S*. *maltophilia* R551-3)	G***GTAG***CGCCGGGCCATGCCCGGCG
NO^*^	**SM11**	YP_004793143 (*S*. *maltophilia* JV3)	T***GTAG***AGTCGAGCCATGCTCGACT
NO^*^	**SM12**	n.a.	***GTAG***AGTCGAGCTTGCTCGACT
NO^*^	**SM13**	n.a.	***GTAG***AGCCGACCGTTGGTCGGCT

REP sequences are denoted in 5´ to 3´ orientation as follows: conserved tetranucleotide in bold and italics, complementary (palindromic) nucleotides underlined, variable nucleotides (IUPAC code) in lower case.

^*^ NO – no orthologous RAYT genes flanked by differing REPs were detected.

n. a. – protein not annotated (see Additional file 1 for these sequences).

### Variability of REP copy numbers

The copy numbers of particular REP element classes were determined and compared in genomes of related bacterial strains. Table 3 and Table 4 reveal a strikingly uneven distribution of REP sequences among different hosts. High REP abundance was found to be restricted to single strain (PF1 and PF22 in *P*. *fluorescens* SBW25, SM13 in *S*. *maltophilia* PML 168), single clade (PF3 in clade B, PF4 in clade H) or several clades (PF8 in clades G and I, PF21 in clades C and H). Various other patterns in distribution can also be detected. REP numbers typically reach hundreds of occurrences of particular REP classes, and are typically more numerous in fluorescent pseudomonads. Here, in four cases, REP numbers exceed a thousand of copies per genome (PF9 in *P*. *sp*. GM48, *P*. *sp*. GM79 and *P*. *fluorescens* R124 and PF10 in *P*. *fluorescens* NZ011). Typically, several REP classes occur in a single host strain.

**Table 3:** The abundances of 22 REP classes in genomes of 63 sequenced fluorescent pseudomonads

**Bacterial strain**	**Clade**	**REP copy number**																					
		Ortho group I^*^		Ortho group II^*^		Ortho group III^*^			Ortho group IV^*^									NO^*^	NO^*^	NO^*^	NO^*^	NO^*^	NO^*^
		**PF 1**	**PF 2**	**PF 3**	**PF 4**	**PF 5**	**PF 6**	**PF 7**	**PF 8**	**PF 9**	**PF 10**	**PF 11**	**PF 12**	**PF 13**	**PF 14**	**PF 15**	**PF 16**	**PF 17**	**PF 18**	**PF 19**	**PF 20**	**PF 21**	**PF 22**
*P*. *agarici* NCPPB 2289	**A**	0	0	0	0	0	0	1	0	0	0	0	0	0	0	0	0	0	0	0	0	0	0
*P*. *fuscovaginae* CB98818		0	0	0	7	0	0	1	0	0	1	0	0	0	0	0	0	0	0	0	0	2	0
*P*. *fuscovaginae* UPB0736		0	0	0	6	0	0	1	0	2	1	0	0	0	0	0	0	0	0	0	0	2	0
*P*. *fluorescens* NZI7	**B**	0	0	**319**	0	0	0	0	1	0	13	4	3	1	0	0	0	**40**	46	0	0	0	0
*P*. *fluorescens* Wayne1		0	0	***420***	0	0	0	0	0	0	0	0	0	0	0	0	0	4	35	0	0	0	0
***P*. *protegens *Pf-5**		0	0	***457***	0	0	0	0	0	0	0	0	0	0	0	0	0	1	37	0	0	0	0
*P*. *chlororaphis* GP72	**C**	0	0	1	0	0	0	0	2	0	0	0	0	0	0	0	0	0	**282**	0	0	**258**	0
*P*. *chlororaphis* O6		0	0	1	0	0	0	0	2	0	0	0	0	0	0	0	0	0	269	0	0	**255**	0
*P*. *chlororaphis* 30-84		0	0	0	0	0	0	0	3	0	0	0	0	0	0	0	0	0	**297**	1	0	17	0
*P*. *sp*. GM17		0	0	1	0	0	0	0	4	0	3	0	0	0	0	0	0	0	**198**	3	0	194	0
*P*. *fluorescens* BBc6R8	**D**	0	0	3	0	0	3	1	0	**739**	3	0	110	5	0	0	0	155	**67**	0	0	0	0
*P*. *sp*. Ag1		0	0	4	0	0	**13**	0	0	**787**	0	0	96	4	0	0	0	154	**66**	0	0	0	0
*P*. *sp*. PAMC 26793		0	0	0	0	0	1	0	0	**684**	2	0	106	4	0	0	0	155	76	0	0	0	0
*P*. *sp*. PAMC 25886		0	0	8	1	0	72	0	0	**32**	0	0	425	14	3	0	0	**117**	20	0	0	0	39
***P*. *fluorescens *A506**		15	0	0	0	0	0	0	0	103	0	0	**681**	63	2	0	0	6	62	0	0	0	0
*P*. *fluorescens* SS101		12	1	0	0	0	0	0	0	101	0	0	**627**	47	1	0	0	3	434	0	0	0	0
*P*. *synxantha* BG33R		73	225	0	0	0	0	0	0	15	0	0	**326**	18	0	0	0	5	64	0	0	0	0
*P*. *fluorescens* NZ007		0	0	0	0	0	0	0	1	**22**	0	0	606	70	0	0	0	**54**	0	0	0	0	0
***P*. *fluorescens *SBW25**		**387**	123	0	0	0	0	0	0	6	0	0	43	6	2	0	0	**104**	0	0	0	0	**202**
*P*. *sp*. R81		45	2	3	0	0	0	0	0	**304**	0	0	140	9	2	0	0	0	27	0	0	0	0
*P*. *fluorescens* NZ052		24	2	11	0	0	1	0	0	199	0	2	226	**21**	1	0	0	28	**281**	0	0	0	3
*P*. *tolaasii* 6264		2	0	0	0	0	0	0	0	10	1	0	797	**151**	0	0	0	20	0	0	0	0	0
*P*. *tolaasii* PMS117		4	0	0	0	0	0	0	0	10	8	0	824	143	1	0	0	33	0	0	0	0	0
*P*. *fluorescens* BRIP34879		12	62	0	0	0	0	0	0	21	0	0	**144**	21	0	0	0	0	4	0	0	0	0
*P*. *extremaustralis* 14-3		20	***168***	4	1	0	0	0	0	4	0	0	0	2	0	0	0	3	**201**	0	0	0	0
*P*. *fluorescens* BS2		55	**181**	0	0	0	0	0	0	10	0	0	1	0	0	0	0	1	0	0	0	0	0
*P*. *fluorescens* WH6		9	41	0	0	0	0	1	2	4	0	0	2	0	0	0	0	27	0	0	0	0	17
*P*. *sp*. UK4		0	0	0	0	0	0	0	0	0	0	0	0	0	0	0	0	0	2	0	0	0	0
*P*. *psychrophila* HA-4	**E**	0	0	0	0	0	0	0	1	2	0	0	0	4	1	0	0	0	21	0	9	0	0
*P*. *fragi* A22		0	0	0	0	0	0	0	0	0	0	0	1	17	116	0	0	0	155	0	0	0	0
*P*. *fragi* B25		0	0	0	0	0	0	0	2	17	4	0	67	163	2	0	0	0	0	0	0	0	0
***P*. *fluorescens *Pf0-1**	**F**	0	0	0	0	0	0	0	3	10	10	0	0	14	31	7	0	0	0	0	0	0	0
*P*. *sp*. GM25		0	0	0	0	0	0	0	29	27	66	0	0	4	9	24	8	0	0	0	0	0	0
*P*. *sp*. R62		0	0	0	0	**150**	0	0	0	5	**832**	7	0	43	145	0	2	0	6	0	99	0	0
*P*. *sp*. GM30		0	0	0	0	**139**	0	0	3	51	**582**	249	0	19	178	7	0	380	13	0	97	0	0
*P*. *fluorescens* R124		0	0	0	0	37	0	0	1	**1009**	**265**	217	0	1	30	5	2	**261**	12	0	**59**	0	0
*P*. *fluorescens* NZ011		0	0	0	0	0	0	0	2	2	**1035**	240	0	0	6	4	2	2	12	0	111	0	0
*P*. *sp*. GM16		0	0	0	0	0	0	0	0	0	159	272	1	14	**357**	2	8	2	9	0	**323**	0	0
*P*. *sp*. GM24		0	0	0	0	0	0	0	0	0	153	261	1	14	**325**	2	8	2	9	0	**304**	0	0
*P*. *sp*. GM80		0	0	0	0	**306**	0	0	0	1	9	182	0	88	***535***	2	0	4	11	2	0	0	0
***P*. *sp*. UW4**	**G**	0	0	0	0	0	0	84	**427**	363	**435**	0	14	60	48	0	0	2	**398**	0	25	0	0
*P*. *sp*. GM33		0	0	0	1	0	0	92	438	540	**156**	0	7	34	21	0	0	***58***	**272**	0	**145**	0	0
*P*. *sp*. GM48		0	0	1	1	0	0	64	48	**1283**	8	0	1	5	0	0	0	**212**	**233**	0	1	0	0
*P*. *sp*. GM49		0	0	1	0	0	0	**108**	**550**	151	47	1	4	79	3	0	0	3	**502**	0	2	0	0
*P*. *sp*. GM55		0	0	**391**	90	0	0	1	33	15	45	0	4	***203***	35	0	0	1	**435**	0	0	1	0
*P*. *sp*. GM74		0	0	1	0	0	0	0	232	428	11	0	2	120	**55**	0	0	19	50	0	19	0	0
*P*. *sp*. GM78		0	0	17	0	0	0	0	9	12	101	92	1	188	**114**	**117**	7	0	150	12	70	53	0
*P*. *fluorescens* NCIMB 11764	**H**	0	0	2	2	51	0	1	2	13	11	0	1	11	5	59	31	**186**	2	0	12	2	0
*P*. *mandelii* JR-1		0	0	0	1	2	0	2	6	5	166	**84**	0	5	2	3	8	164	68	0	0	**355**	0
*P*. *fluorescens* HK44		0	0	0	0	0	0	56	10	5	29	0	1	0	0	0	0	**304**	1	100	1	1	0
*P*. *sp*. GM50		0	0	0	0	3	0	**392**	1	**198**	32	0	4	20	0	15	11	13	4	0	67	**667**	0
*P*. *sp*. GM102		0	0	0	0	0	0	140	2	77	6	0	16	**116**	1	11	14	**245**	0	0	57	**679**	0
*P*. *sp*. GM79		0	0	1	400	0	0	16	2	**1417**	8	0	9	62	0	0	0	127	0	0	168	75	0
*P*. *sp*. GM60		0	0	31	**398**	0	0	0	1	***175***	30	50	0	5	3	17	6	0	297	0	1	**379**	0
*P*. *sp*. GM67		0	0	20	**301**	0	0	0	2	33	41	23	0	4	1	37	26	0	101	1	0	**656**	0
*P*. *sp*. GM21		0	0	3	0	1	0	0	12	48	147	20	4	35	4	38	**9**	0	11	0	0	0	0
*P*. *sp*. GM18		0	0	0	0	0	0	27	1	2	3	0	1	7	0	0	0	28	1	1	65	35	0
*P*. *sp*. GM41(2012)		0	0	0	1	55	0	73	7	72	91	13	2	13	0	**19**	5	0	5	**174**	89	24	0
*P*. *fluorescens* Q2-87	**I**	0	0	0	0	47	0	0	75	**576**	130	0	0	2	0	0	0	**586**	2	1	0	0	0
***P*. *fluorescens *F113**		0	0	0	0	**331**	0	0	**749**	60	91	0	0	0	0	0	0	54	3	198	9	0	0
*P*. *fluorescens* Q8r1-96		0	0	0	0	30	0	0	**661**	61	109	0	0	0	0	0	0	44	5	**295**	17	0	0
*P*. *fluorescens* Wood1R		0	0	0	0	21	0	0	**290**	26	62	0	0	1	0	0	0	37	6	181	15	0	0
***P*. *brassicacearum *NFM421**		0	0	0	0	23	0	0	**632**	60	116	0	0	1	0	0	0	46	6	**303**	14	0	0

The values represent total numbers of REP sequences from Table 1 in different strains´ genomes. The values are denoted in bold and underlined in cases where RAYT gene associated with a particular REP class is present in the genome. Presence of pseudogenized RAYT genes, containing nonsense or frameshift mutations or deletions, is denoted by italics. The phylogenetic groups are marked with letters A to G as in Figure 1. The names of strains whose complete genomic sequences were determined are in bold.

^*^ - as in Table 1.

**Table 4:** The abundances of 13 REP classes in genomes of 10 sequenced stenotrophomonads

**Bacterial strain**	**Clade**	**REP copy number**												
		Ortho group I^*^			Ortho group II^*^		Ortho group III^*^		NO^*^	NO^*^	NO^*^	NO^*^	NO^*^	NO^*^
		**SM**	**SM**	**SM**	**SM**	**SM**	**SM**	**SM**	**SM**	**SM**	**SM**	**SM**	**SM**	**SM**
		**1**	**2**	**3**	**4**	**5**	**6**	**7**	**8**	**9**	**10**	**11**	**12**	**13**
*S*. *maltophilia* PML168	**A**	1	0	0	5	**18**	0	0	37	0	2	1	0	**96**
*S*. *maltophilia* S028		0	0	0	0	0	0	**45**	3	0	4	0	0	4
***S*. *maltophilia *R551-3**	**no**	**39**	4	16	62	1	6	0	266	**49**	**259**	49	18	0
*S*. *sp*. SKA-14	**no**	7	**37**	12	**128**	1	0	0	**323**	3	7	31	**82**	2
***S*. *maltophilia *D457**	**B**	31	8	**18**	37	2	3	0	**258**	***92***	5	15	0	2
***S*. *maltophilia *JV3**		**57**	4	7	**183**	2	1	0	**283**	9	10	**108**	1	1
*S*. *maltophilia* RR-10	**C**	18	**8**	10	98	0	6	0	47	**106**	**120**	15	2	0
*P*. *geniculata* N1		***33***	7	10	99	1	**12**	0	61	**116**	**107**	18	1	0
***S*. *maltophilia *K279a**		**52**	16	11	105	2	33	0	**427**	7	13	3	2	1
*S*. *maltophilia* Ab55555		**55**	13	9	***102***	4	**31**	0	**375**	6	12	3	1	1

The values represent total numbers of REP sequences from Table 2 in different strains´ genomes. The values are denoted in bold and underlined in cases where RAYT gene associated with a particular REP class is present in the genome. Presence of pseudogenized RAYT genes, containing nonsense or frameshift mutations or deletions, is denoted by italics. The phylogenetic groups are marked with letters A to C as in Figure 2. The names of strains whose complete genomic sequences were determined are in bold.

^*^ - as in Table 2.

### RAYTs and REP abundance

Finally, we examined if the presence of RAYTs influenced REP abundance. In most cases, RAYTs associated with abundant REP classes were indeed present in host bacterial strains (Table 3, Table 4, Additional file 3, Additional file 4). On average, two to three RAYTs were present per strain. A maximum of four RAYTs were detected in a single host genome, and several strains contained no RAYTs at all. Sometimes, the RAYT genes contained frameshift or nonsense mutations, indicative of recent pseudogenization. Interestingly, three strains (*P*. *fluorescens* R124, *P*. *sp*. UW4 and *P*. *sp*. GM78) contained two RAYTs associated with two different REPs belonging to the same orthogroup IV. In these cases, one RAYT gene is always located at a novel chromosomal site. This indicates different evolutionary origins of these RAYTs/REPs, for example RAYT duplication followed by mutation of flanking REPs into another REP class of orthogroup IV, or horizontal acquisition and integration of RAYT gene into a novel locus.

The instances when particular REPs were overrepresented while their cognate RAYTs were absent appeared quite often. However, for a great majority of these cases, one of the following was also observed: i) related strains possessed RAYTs associated with REP sequences in question, or ii) RAYTs in given strain were associated with different REP classes, belonging to the same orthogroup (Additional file 3, Additional file 4). As for i), this might indicate loss of RAYT genes from host strain. As for ii), this was represented for example by fluorescent pseudomonads of clade D which harboured REP classes PF9, PF12 and PF13 of orthogroup IV. While multiple copies of each of these REP classes were present, RAYT associated with only one class was detected in each genome. From this, it can be inferred that original REP sequences flanking the RAYT genes have undergone mutations into another REP variants and were subsequently multiplicated, leading to the presence of both classes from the same REP orthogroup in host genomes. We will call this process an orthoswitch. Although the assumed orthoswitches occurred considerably frequently (*i*.*e*. at least once in every orthogroup, Table 1 and Table 2), we can only speculate about their molecular mechanism.

In Additional file 5, a model to explain the discrepancies between REP abundance and RAYT presence/absence is proposed. The model assumes an active role of RAYTs in REP proliferation, based on their REP-dependent nuclease activity [23] and coupling of transcription and translation in uncompartmentalized bacterial cell, allowing for preferential RAYT action on REPs that flank their encoding genes (due to their juxtaposition during RAYT expression). According to the model, only the presence of an active RAYT can support multiplication of its cognate REPs and their long-term persistence. When RAYT is inactivated by pseudogenization or completely lost from the host genome, the cognate REPs could no longer multiply, leading to their gradual degradation by mutational processes (Additional file 5A). Depending on when RAYT loss/inactivation occurred, corresponding numbers of REP elements would remain in the host chromosome. Similarly, if an orthoswitch occurred, novel REP variants associated with RAYT genes would spread, while the original REP elements would remain in the host genome and decay mutationally (Additional file 5C). Furthermore, RAYT duplication with concomitant REP diversification (which could proceed with mechanism similar to orthoswitch, see above) would lead to emergence of novel REP classes (Additional file 5B). Finally, horizontal transfer from closely or more distantly related strains might have significantly impacted the REP/RAYT diversity within the analyzed genomes. Horizontal transfer is likely to have accounted at least for the isolated occurrences of some RAYTs and their cognate REPs (for example PF3 in *P*. *sp*. GM55, see Table 3).

## Conclusions

In the last decade, there has been a considerable resurgence of interest in REP elements. This was prompted by several factors, notably genomic analyses of newly sequenced bacteria which revealed novel REP elements [29], and the discovery of candidate REP mobilizers, RAYTs [14]. In this study, we aimed to assess the diversity of REP elements and RAYTs in large genomic sets of environmental bacteria – fluorescent pseudomonads and stenotrophomonads. Two previous works have already focused on the intraspecific variability of REPs [12,13], but their authors used different, less stringent criteria for REP selection leading to a more relaxed definition of REP classes. We analyzed precisely those REP elements for which association with RAYTs was detected. In addition, our dataset was much broader than those of the two aforementioned studies [12,13]. Our results confirm that REPs of fluorescent pseudomonads and stenotrophomonads are very diverse and dynamic. Also, REP host specificity ranges greatly: strain-specific, clade ("subspecies")-specific and species-specific REP sequences were observed (Table 3, Table 4).

Such large-scale analysis of diverse bacteria allowed us to reconstruct the evolutionary scenario for these repetitive elements and associated RAYTs. Since RAYTs of both bacterial groups are monophyletic (Additional file 2), unique original RAYTs were likely to be present in the genomes of common ancestors of fluorescent pseudomonads and stenotrophomonads, their genes flanked by ancestral REPs. Later during evolution, RAYT genes have undergone duplications and diversified to the state which is seen in more derived clades (Table 3, Table 4), with concomitant diversification of their cognate REPs. The later the novel RAYT/REP variants emerged, the more phylogenetically restricted incidence they show. Novel REP variants might also partially replace the original ones following an orthoswitch (Additional file 5). Upon RAYT pseudogenization which may be followed by RAYT loss from the host genome, proliferation of cognate REPs would cease. Although beneficial roles for REPs have been proposed (see Background), extremely high REP numbers might pose a burden to bacterial hosts, and RAYT inactivation could help keep REP numbers within range tolerable by host cell. A minority of derived strains would lose all RAYTs, leading to greatly reduced REP numbers in their genomes (Table 3).

Since the mechanisms behind REP dissemination and changeability are not known yet, our findings could provide foundations for understanding the evolution of REP element diversity and suggest possible directions for further laboratory research.

## Methods

### Genomic analyses

Bacterial genomic sequences were downloaded from the NCBI Genome database [30]. RAYTs were identified by performing TBLASTN search [31], using previously described *Pseudomonas fluorescens* and *Stenotrophomonas maltophilia* RAYTs [14] as query sequences. RAYTs that were not annotated were conceptually translated from corresponding DNA sequences using Transeq [32]. Identified RAYTs were checked to contain previously characterized sequence motifs peculiar to RAYTs [14]. REP sequences flanking *rayt* genes were identified as inverted repeats located both upstream and downstream of the genes, with characteristic REP features: conserved 5´-terminal tetranucleotide (GT^A^/_G_G) and downstream palindrome. REP copy numbers were determined using pDRAW32 [33].

### Phylogenetic analyses

Concatenated complete nucleotide sequences of genes coding for RNA polymerase beta subunit (*rpoB*), DNA gyrase beta subunit (*gyrB*) and RNA polymerase sigma subunit (*rpoD*) as well as RAYT protein sequences were processed with MEGA5 package [34]. Sequences were aligned, trimmed of unaligned nucleotides or amino acids, and Neighbor-Joining phylograms were constructed with 1 000 bootstrap replicates.

## Competing interests

The authors declare that they have no competing interests.

## Authors´ contributions

JN conceived the study, performed the analysis and drafted the manuscript. BS and IL supervised the work and critically read the manuscript. All authors read and approved the final manuscript.

## Supplementary Material

Additional file 1 **Nucleotide sequences of unannotated *rayt *genes and amino acid sequences of translated RAYTs.** The RAYT symbols are as in Table 1 and Table 2. Click here for file

Additional file 2 **Neighbor-Joining phylogram of representative selection of RAYT proteins across the domain *Bacteria. ***RAYTs of fluorescent pseudomonads and stenotrophomonads are denoted by their symbols as in Table 1 and Table 2. The remaining RAYTs are denoted by their accession numbers and host strain names. All included RAYTs share unique motifs in their sequences and are flanked by inverted repeats with characteristics of REPs (conserved 5´-terminal tetranucleotide and downstream palindromic region). Red circles denote the origins of lineages specific for fluorescent pseudomonads and stenotrophomonads, respectively. Click here for file

Additional file 3 Sorting of REP copy numbers in fluorescent pseudomonads based on their association with RAYTs. Click here for file

Additional file 4 Sorting of REP copy numbers in stenotrophomonads based on their association with RAYTs. Click here for file

Additional file 5 **A model of long-term evolution of REP elements in genomes of fluorescent pseudomonads and stenotrophomonads.** The proposed events are: **A** – recent (left) and ancient (right) RAYT loss, **B** – recent (left) and ancient (right) RAYT duplication and diversification, **C** – recent (left) and ancient (right) REP orthoswitch. REP elements (dots) and their associated *rayt* genes (arrows) are denoted with the same color. The host chromosomes are represented as black circles. The REP numbers are approximate.The model is based on two premises: i) RAYTs specifically disseminate their cognate REP elements throughout their host genome, ii) REP copies are getting slowly but constantly lost from host genome, due to accumulation of mutations. See Results and Discussion for further explanation. Click here for file
