# Supplementary material for: Evolution of REP diversity: a comparative study
Source: BMC Genomics. 2013 Jun 10;14:385. doi: 10.1186/1471-2164-14-385 (PMC3686654; doi:10.1186/1471-2164-14-385)
Supplement: Additional file 1 — Nucleotide sequences of unannotated rayt genes and amino acid sequences of translated RAYTs. The RAYT symbols are as in Table 1 and Table 2. [file 1471-2164-14-385-S1.pdf]

PF2

>gb|AMZG01000009.1|:c100096-99641 Pseudomonas fluorescens BS2 Contig_9, whole genome shotgun sequence
ATGCCTGATCTACCCGCTTCACATCGCCTACGCATCGGTCGATTCAGTGAGCCGAACCGCATCTACCTCA
TCACCACCAACACCCACGAACGGATGCCGATATTCGGTGATTTCCAGCTCGGGCGATTAGTCGTCCGGCA
GTTTCGATTGGCCCAGTACCAACGGCTGGCAAATACCCTGGCATGGGTAATCATGCCGGATCACTTTCAT
TGGATTGTTGAGCTGCAAAAAGGCTCACTGGCCAGCCTGATGTGCCAGGTCAAGTCCAAAAGCACGCGCG
CCGTCAATGGTGCAAGCGGTCGCAAGGGGCGGCTTTGGCAGGCGAGCTTCCATGACCGGGCACTGCGCAA
GGAGGATGACCTGGTGAAAATGGCCAGGTATGTGGTGGCCAATCCCTTGCGAGCGGGGCTCGTGAAGCGA
ATCGGCGACTATCCACTGTGGGATGCCATCTGGTTG
>PF2 Pseudomonas fluorescens BS2
MPDLPASHRLRIGRFSEPNRIYLITTNTHERMPIFGDFQLGRLVVRQFRLAQYQRLANTL
AWVIMPDHFHWIVELQKGSLASLMCQVKSKSTRAVNGASGRKGRLWQASFHDRALRKEDD
LVKMARYVVANPLRAGLVKRIGDYPLWDAIWL

PF3

>gb|AJXF01000173.1|:5369-5833 Pseudomonas fluorescens NZI7 contig00183_contig_1, whole genome shotgun sequence
ATGCTCCAGCGTTTTCACGGCAACCGGCTGCGTACCGGGCGCTACTCGCAAACCGGCCAGATCTACCTGC
TCACCGCGGTGACCCACTGTCGTGAGCGGTGTTTCGACGATTGGCGCATCGGCCGCCTGCTGGTGTGGCA
ACTGCGTCAGGCCCAGCGGCAAGGGTTGGCCCATTCCCTGGCCTGGGTGGTGATGCCGGATCATCTTCAC
TGGCTGCTGGAACTGGGCGGTTGCTCATTGGCGGCCCTGATGCGCCGGATCAAGTCCTGTAGCGCACGGG
AGATCAACACCCACCTGCAACGCAGCGGTCGGCTTTGGCAAAAGGGTTACCACGACCGCGCCCTGCGTCG
TGACGAGGACCTGCGCGTGGTGGCCCGCTACCTTATCGCCAACCCGATTCGCGCCGGCCTTGTCACCCGC
GTGCACGACTACCCTCTGTGGGACGCCGTGTGGCTGAACGATTCC
>PF3 Pseudomonas fluorescens NZI7
MLQRFHGNRLRTGRYSQTGQIYLLTAVTHCRERCFDDWRIGRLLVWQLRQAQRQGLAHSL
AWVVMPDHLHWLLELGGCSLAALMRRIKSCSAREINTHLQRSGRLWQKGYHDRALRRDED
LRVVARYLIANPIRAGLVTRVHDYPLWDAVWLNDS

PF6

>gb|AKVH01000100.1|:63395-63892 Pseudomonas sp. Ag1 Contig6, whole genome shotgun sequence
CTGCTAACGTCAATTGACTACTTCCCCTCAAGGAGCAAAGGAATACTTCATCAACCCAACTCTCATCTGT
TACGCCGTGGTCGACATTCAGAATCTGGCCGTCCTTATCTGATTACCGCCGTGGTTCACAACCGGCAACC
CCTGTTCAAAGACTTCCATTTGGGAAGACTACTGGTAGCAGAATTCAGGAAGACCCATGAATTGGGAATG
GTCGACTCTCTGGCATGGGTCGTCATGCCTGATCACTTTCATTGGCTATTTGAGCTGCAAGGCACAACGT
TGTCGCAGGCTGTGGGACGTACCAAATCCCGCAGCACCCTCACCATTAATCGCGCACGCGGCAGCAAAGA
ACGATTCTGGCAACTTGGCTACCATGACCGAGCAGTGCGTAGCGATGAAGACCTTCGCAAAATTGCCCGC
TACATCATCGCCAATCCTCTGCGAGCGGGCCTGGTCGAACATATTGGTGACTACCCACTATGGGACGCAG
CCTGGCTC
>PF6 Pseudomonas sp. Ag1
MLTSIDYFPSRSKGILHQPNSHLLRRGRHSESGRPYLITAVVHNRQPLFKDFHLGRLLVA
EFRKTHELGMVDSLAWVVMPDHFHWLFELQGTTLSQAVGRTKSRSTLTINRARGSKERFW
QLGYHDRAVRSDEDLRKIARYIIANPLRAGLVEHIGDYPLWDAAWL

PF11

>gb|AJFM01000007.1|:c222739-222284 Pseudomonas mandelii JR-1 contig00007, whole genome shotgun sequence
ATGCCGGATTTACCTGCTGCACACCGATTGCGAGTCGGGCGCTATGCCGAAGCCAGCCGGATTTATTTGT
TAACCAGCAATACACTTCACAGGATCCCGGTTTTCAAGGATTTTGCCTTGGGCAGGCTGGTCGTCGATCA
ATTTCGAAATGCGCAGAATCTAGGCTTGGCGAACTCACTGGCGTGGGTAGTCATGCCCGATCATTTTCAT
TGGTTGATTGAATTACAGCAGGGCTCTTTGAGCGAGTTGATGCAAAAGACCAAGTCGATGAGTACCAAAG
CGGTGAAGCAGCGTACCGGTCGAAATATTAGCCTCTGGCAGAGAGGATTCCATGATCGGGCACTTCGGCG
GGAGGAGGATTTGGTGAAGTTGGCGAGGTATGTCGTGGCCAACCCGTTGCGGGCTGGACTGGTGGAGAAA
CTGGGCGATTATCCGTTGTGGGATGCGATTTGGGTT

>PF11 Pseudomonas mandelii JR-1
MPDLPAAHRLRVGRYAEASRIYLLTSNTLHRIPVFKDFALGRLVVDQFRNAQNLGLANSL
AWVVMPDHFHWLIELQQGSLSELMQKTKSMSTKAVKQRTGRNISLWQRGFHDRALRREED
LVKLARYVVANPLRAGLVEKLGDYPLWDAIWV

PF13

>gb|AKJB01000001.1|:81106-81561 Pseudomonas sp. GM102 PMI18_contig_3.3, whole genome shotgun sequence
TTGCCTGATCTTCCTGCTTCACATCGCCTACGAACGGGGCGCTATGCCGAACCCAATCGAATCTACTTAC
TGACTACCAATACACTTGATCGCGAGCCGGTATTTGCGGATTTCGCATTGGGCAGGTTGGTTGTTCATCA
ATTTCGCCAAGCACAGAACACAGGGTTAGTGAATTCACTGGCATGGGTGGTCATGCCTGATCACTTCCAT
TGGCTTGTCGAATTGGAGAAATGTTCGCTCAGTAATCTGATGCGTCAAACCAAGTCACTGATTACGCGGG
AGGTGAATCTTTCCAGTAACAGGAACGGACCACTTTGGCAGCAGGGCTATCACGACCGGGCATTGAGGCG
AGAAGAAGACTTGGTGAAGATGGCACGGTATGTAGTGGCCAATCCATTACGGGCAGGTCTTGTAGAGCGG
CTTGGCGACTATCCGTTGTGGGATGCCATCTGGCTT
>PF13 Pseudomonas sp. GM102
LPDLPASHRLRTGRYAEPNRIYLLTTNTLDREPVFADFALGRLVVHQFRQAQNTGLVNSL
AWVVMPDHFHWLVELEKCSLSNLMRQTKSLITREVNLSSNRNGPLWQQGYHDRALRREED
LVKMARYVVANPLRAGLVERLGDYPLWDAIWL

SM5

>gi|390189365:71523-71975 Stenotrophomonas maltophilia PML168 WGS project CAJH00000000 data, contig NODE_PML168_22_len_256555_cov_67_936806C1, whole genome shotgun sequence
ATGGCCAGCCAACGCCTCCAACGCGGCCGATACTCGCATCCAGGGTATTGCTACGCGCTCACCACCACCA
CGCACGACAGGCGCAAGTGGTTCGAGGATCCCCTCAATGCAGAGGTAATCATCGACACGCTGCGCTACAT
GGATCGTTGCGGGGTCAGTTGCACGTTAGCTTGGGTGGTAATGCCGGACCACGTGCATTGGTTGATCCAA
TTGCGGCAAGACACCTTGGCTCGTTGCATGCTGCTGTTCAAATCACGCAGCAGCCGATTGTTGAATGAGC
GTCTGCAGCGAAAGGGAAAGCTCTGGCAGCACGGGTACTTCGACCACGCCGTGAGAAATGAGGCATCCTT
GCGCCGGCAGGCGCTGTACATCCTCGCCAACCCGATACGTGCGGGATTGGCCAGTGCGTTGGGGGAGTAC
CCGCACGCCTGGTCGCGGTGGCCCATGGAAGCG
>SM5 Stenotrophomonas maltophilia PML168
MASQRLQRGRYSHPGYCYALTTTTHDRRKWFEDPLNAEVIIDTLRYMDRCGVSCTLAWVV
MPDHVHWLIQLRQDTLARCMLLFKSRSSRLLNERLQRKGKLWQHGYFDHAVRNEASLRRQ
ALYILANPIRAGLASALGEYPHAWSRWPMEA

SM6

>gb|ALOG01000013.1|:c378830-378351 Stenotrophomonas maltophilia Ab55555 cont1.13, whole genome shotgun sequence
ATGCCCAGCCCTCAACTGCTTGCCGGCCGCCGATCCATAGTCGGCAACGTCTACACCATCACCATGGTGT
GCCGGAACCGCCATCGTGTTTTCGATAGCCCTGCCAATGCCGACCTTGCCATGCAGCTCCTCGGATCGAT
GGATCGGGAAGGCCTGACTGCATCGTTTGCCTGGGTGATCATGCCGGATCACATCCACTGGCTGGCTCAA
CTTCGTGGCCATTCGCTGGGCTACTGCGTGCAGCGCTTCAAGGCGCGCAGCAGCTTTCTAATCAACCGGC
GGCGAGGGAGCCAGGGTGCAATCTGGCAGGCGGGTTATCACGATCATGCGATCCGCAGTGACGCGTCGCT
GCACAGGCACGCTTGCTACATTCTGGCAAATCCCGTTCGAGCCGGTCTTGCCGCGCAGATCGGTGACCAT
CCGTACGGATGGTGCCGCTGGCCGTTGAGCGAGCTTGAGTCGGCAGGCGAACGCGCATGG
>SM6 Stenotrophomonas maltophilia Ab55555
MPSPQLLAGRRSIVGNVYTITMVCRNRHRVFDSPANADLAMQLLGSMDREGLTASFAWVI
MPDHIHWLAQLRGHSLGYCVQRFKARSSFLINRRRGSQGAIWQAGYHDHAIRSDASLHRH
ACYILANPVRAGLAAQIGDHPYGWCRWPLSELESAGERAW

SM7

>gb|ALYK01000218.1|:c15553-15104 Stenotrophomonas maltophilia S028 scaffold73_5, whole genome shotgun sequence
ATGGCGAGTCCTTCTCTACAACGCGGACGCATCTCCTGCGAGGGCAGCTACTACGTCGTCACCACGGTTA

```
CGGCGCAGCGCACCCGCCTGTTCGTCGATACCGCCAATGCGCAGGAGGTCTGCCACTGGCTGCGCACCTC
GGATGCCCAAGGCCGCACCGACTCCCTGACATGGGTGGTCATGCCCGACCATATCCACTGGATTTTCCGC
CTGCGCGATACACCACTGTCGATCGTGATGCGAACCTTCAAGTCGCGGTCGGCCAAGGCCCTCAACCAGA
GCAACAGCACCCGGGGCACCGTTTGGCAACCGGGCTACTACGACCAGCTGCAGCGCGACGATCGCCATCT
GTTGGCCGCGGCCACCTACATACTCGCCAACCCGCTGCGCGCCGGATTGGCCAAGGGCATGAACGATTAC
CCCTTCGCCTGGTGCCGATGGCCCCTCCCG
>SM7 Stenotrophomonas maltophilia S028
MASPSLQRGRISCEGSYYVVTTVTAQRTRLFVDTANAQEVCHWLRTSDAQGRTDSLTWVV
MPDHIHWIFRLRDTPLSIVMRTFKSRSAKALNQSNSTRGTVWQPGYYDQLQRDDRHLLAA
ATYILANPLRAGLAKGMNDYPFAWCRWPLP
```

SM12

```
>gb|ACDV01000017.1|:114731-115180 Stenotrophomonas sp. SKA14
ctg_1108481805189, whole genome shotgun sequence
ATGCCCAGTCCAGCCTTGTTGCGCGGCCGATGCTCCACGGTCGGATGCTTCTACGTAGTCACCACTGTCA
CTGCCGGACGCCAACGCGTCTTTGCAGATCCGCGATGCGTGGATGCCTTGACTGATGAGCTTCGAGGCAG
CGACTGCAAGGGGATCACGCGGTCATTGGCTTGGGTGGTGATGCCGGATCATCTGCACTGGGTTCTGCAG
TTGCGCGAAGGTACGCTTGGCCGCTGCATGCCATCACTCAAGTCCAGGGTGGCGATCGCGGTCAACGCGC
ACTCCGGTGAAGGACGCCCGGTGTGGCAGCGGGGCTACTACGATCACCTGATCCGGAATGAGGAGGATGT
CCGACAACAAGCGCTGTATGCCATGGCCAACCCTGTACGCGCGGGTCTGGCGTCAACCCTAGGGGAGTAT
CCATTCGCATGGTGTTGCTGGCCGCTCGAA
>SM12 Stenotrophomonas sp. SKA14
MPSPALLRGRCSTVGCFYVVTTVTAGRQRVFADPRCVDALTDELRGSDCKGITRSLAWVV
MPDHLHWVLQLREGTLGRCMPSLKSRVAIAVNAHSGEGRPVWQRGYYDHLIRNEEDVRQQ
ALYAMANPVRAGLASTLGEYPFAWCCWPLE
```

SM13

```
>gi|390189362:13302-13766 Stenotrophomonas maltophilia PML168 WGS project
CAJH00000000 data, contig NODE_PML168_62_len_243821_cov_47_250263C1, whole
genome shotgun sequence
ATGCCCAGCCCACGCCTTCATCACGGCCGCCACTCCCGCATCGACACCGTGTACGCGCTGACGACCATCA
CGCATGCGCGTGTACCCCATTTCCACCAGCCCGACCTGGCCAGGCATGTCATGCAGGTGCTGCATGCCAT
GGAAGACGAGCATCGGGTCTGCAACCTTGCGTGGGTGGTCATGCCAGACCACGTGCATTGGCTGATGCAG
CTCCGGCAGGGCTCCCTGGGCGCATGCCTGCAGCGCTTCAAATCGCGCAGCAGCCTGCTCATCAATCGCC
AATCCGCCCGCAACGGCCCTGTCTGGCAAGCCGGTTATTACGACCACGCACTACGCAAGGACGAAGACCT
GCGACGCCAAGCCACCTACATCCTCGCCAACCCGGTCCGTGCCGGCCTGGCGGAGGTGATCGGTGCGTAT
CCCTTCTCGTGGTGCAGATGGCCCACCGAAGACCATCCGCCCGGG
>SM13 Stenotrophomonas maltophilia PML168
MPSPRLHHGRHSRIDTVYALTTITHARVPHFHQPDLARHVMQVLHAMEDEHRVCNLAWVV
MPDHVHWLMQLRQGSLGACLQRFKSRSSLLINRQSARNGPVWQAGYYDHALRKDEDLRRQ
ATYILANPVRAGLAEVIGAYPFSWCRWPTEDHPPG
```
