# Supplementary figures and images for: Evolution of REP diversity: a comparative study
Source: BMC Genomics. 2013 Jun 10;14:385. doi: 10.1186/1471-2164-14-385 (PMC3686654; doi:10.1186/1471-2164-14-385)

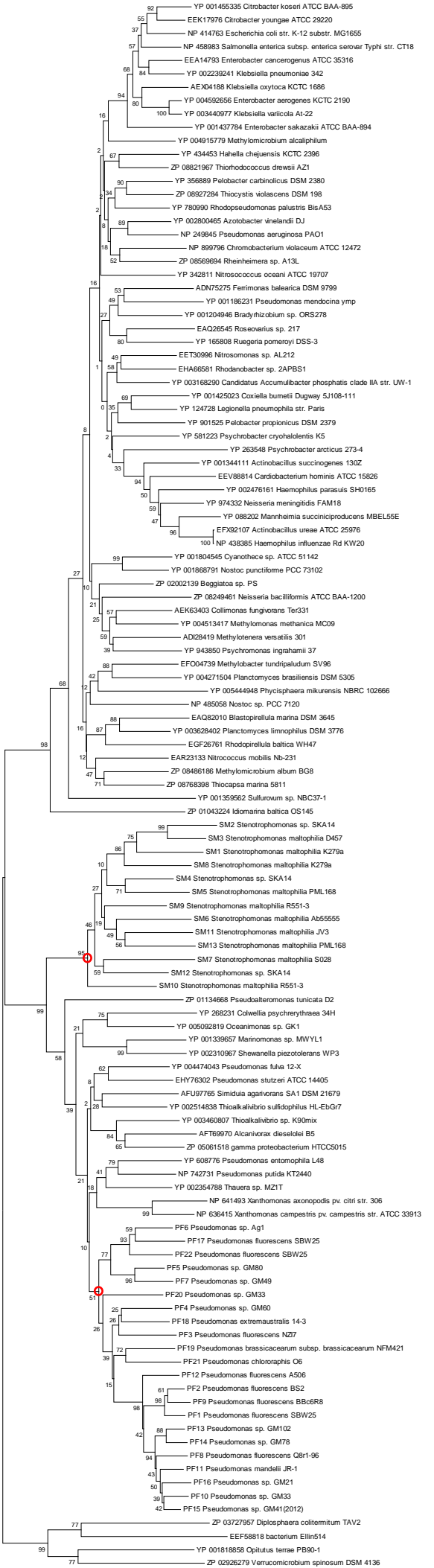

Supplement: Additional file 2 — Neighbor-Joining phylogram of representative selection of RAYT proteins across the domain Bacteria. RAYTs of fluorescent pseudomonads and stenotrophomonads are denoted by their symbols as in Table 1 and Table 2. The remaining RAYTs are denoted by their accession numbers and host strain names. All included RAYTs share unique motifs in their sequences and are flanked by inverted repeats with characteristics of REPs (conserved 5´-terminal tetranucleotide and downstream palindromic region). Red circles denote the origins of lineages specific for fluorescent pseudomonads and stenotrophomonads, respectively. [file 1471-2164-14-385-S2.pdf]

A

B

C

evolutionary time

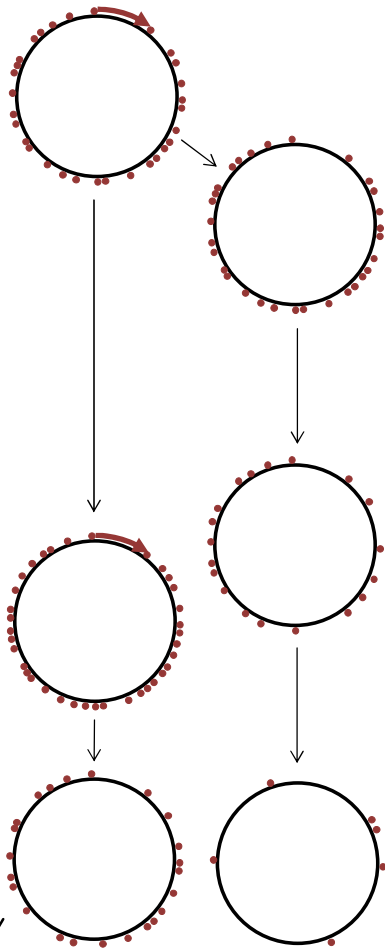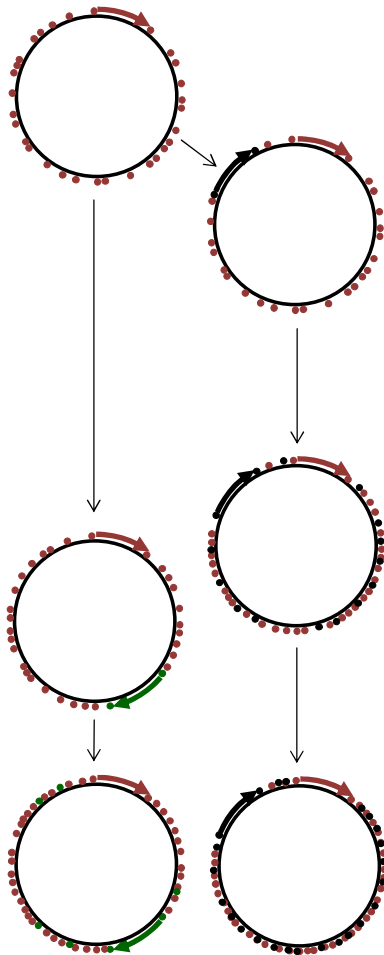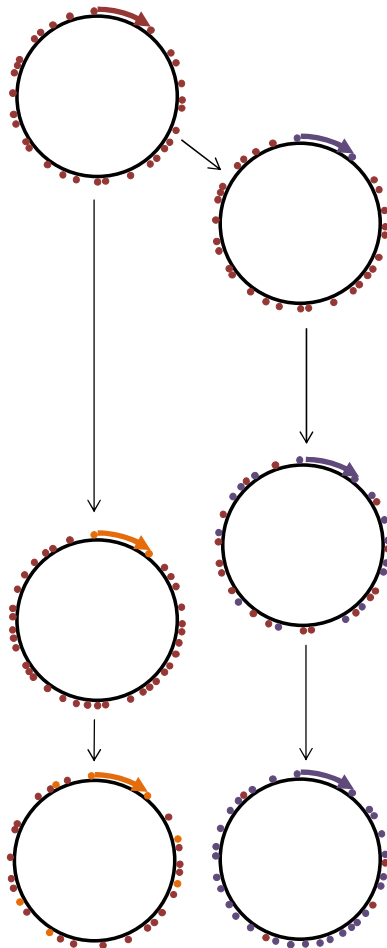

Supplement: Additional file 5 — A model of long-term evolution of REP elements in genomes of fluorescent pseudomonads and stenotrophomonads. The proposed events are: A – recent (left) and ancient (right) RAYT loss, B – recent (left) and ancient (right) RAYT duplication and diversification, C – recent (left) and ancient (right) REP orthoswitch. REP elements (dots) and their associated rayt genes (arrows) are denoted with the same color. The host chromosomes are represented as black circles. The REP numbers are approximate.The model is based on two premises: i) RAYTs specifically disseminate their cognate REP elements throughout their host genome, ii) REP copies are getting slowly but constantly lost from host genome, due to accumulation of mutations. See Results and Discussion for further explanation. [file 1471-2164-14-385-S5.pdf]
