# Supplementary material for: Evolution of REP diversity: a comparative study
Source: BMC Genomics. 2013 Jun 10;14:385. doi: 10.1186/1471-2164-14-385 (PMC3686654; doi:10.1186/1471-2164-14-385)
Supplement: Additional file 3 — Sorting of REP copy numbers in fluorescent pseudomonads based on their association with RAYTs. [file 1471-2164-14-385-S3.pdf]

Each field in the following table and each point in the following graph correspond to the copy number of particular REP class in host genome (cases of 10 or less REP occurrences per genome were discarded).

The colors mark one of the following conditions:

Green – intact cognate RAYT is present

Blue – pseudogenized cognate RAYT is present

Purple – cognate RAYT is absent, RAYT of the same orthogroup is present, cognate RAYT is present in strains of the same clade

Red - cognate RAYT is absent, RAYT of the same orthogroup is present, cognate RAYT is absent in strains of the same clade

Orange - cognate RAYT is absent, RAYT of the same orthogroup is absent, cognate RAYT is present in strains of the same clade

Grey - cognate RAYT is absent, RAYT of the same orthogroup is absent, cognate RAYT is absent in strains of the same clade

Bacterial strain	Clade	REP copy number																					
		Ortho group I		Ortho group II		Ortho group III			Ortho group IV									NO*	NO*	NO*	NO*	NO*	NO*
		PF 1	PF 2	PF 3	PF 4	PF 5	PF 6	PF 7	PF 8	PF 9	PF 10	PF 11	PF 12	PF 13	PF 14	PF 15	PF 16	PF 17	PF 18	PF 19	PF 20	PF 21	PF 22
P. agarici NCPPB 2289	A	0	0	0	0	0	0	1	0	0	0	0	0	0	0	0	0	0	0	0	0	0	0
P. fuscovaginae CB98818		0	0	0	7	0	0	1	0	0	1	0	0	0	0	0	0	0	0	0	0	2	0
P. fuscovaginae UPB0736		0	0	0	6	0	0	1	0	2	1	0	0	0	0	0	0	0	0	0	0	2	0
P. fluorescens NZI7	B	0	0	319	0	0	0	0	1	0	13	4	3	1	0	0	0	40	46	0	0	0	0
P. fluorescens Wayne1		0	0	420	0	0	0	0	0	0	0	0	0	0	0	0	0	4	35	0	0	0	0
***P. protegens* Pf-5**		0	0	457	0	0	0	0	0	0	0	0	0	0	0	0	0	1	37	0	0	0	0
P. chlororaphis GP72	C	0	0	1	0	0	0	0	2	0	0	0	0	0	0	0	0	0	282	0	0	258	0
P. chlororaphis O6		0	0	1	0	0	0	0	2	0	0	0	0	0	0	0	0	0	269	0	0	255	0
P. chlororaphis 30-84		0	0	0	0	0	0	0	3	0	0	0	0	0	0	0	0	0	297	1	0	17	0
P. sp. GM17		0	0	1	0	0	0	0	4	0	3	0	0	0	0	0	0	0	198	3	0	194	0
P. fluorescens BBc6R8	D	0	0	3	0	0	3	1	0	739	3	0	110	5	0	0	0	155	67	0	0	0	0
P. sp. Ag1		0	0	4	0	0	13	0	0	787	0	0	96	4	0	0	0	154	66	0	0	0	0
P. sp. PAMC 26793		0	0	0	0	0	1	0	0	684	2	0	106	4	0	0	0	155	76	0	0	0	0
P. sp. PAMC 25886		0	0	8	1	0	72	0	0	32	0	0	425	14	3	0	0	117	20	0	0	0	39
***P. fluorescens* A506**		15	0	0	0	0	0	0	0	103	0	0	681	63	2	0	0	6	62	0	0	0	0
P. fluorescens SS101		12	1	0	0	0	0	0	0	101	0	0	627	47	1	0	0	3	434	0	0	0	0
P. synxantha BG33R		73	225	0	0	0	0	0	0	15	0	0	326	18	0	0	0	5	64	0	0	0	0
P. fluorescens NZ007		0	0	0	0	0	0	0	1	22	0	0	606	70	0	0	0	54	0	0	0	0	0
***P. fluorescens* SBW25**		387	123	0	0	0	0	0	0	6	0	0	43	6	2	0	0	104	0	0	0	0	202
P. sp. R81		45	2	3	0	0	0	0	0	304	0	0	140	9	2	0	0	0	27	0	0	0	0
P. fluorescens NZ052		24	2	11	0	0	1	0	0	199	0	2	226	21	1	0	0	28	281	0	0	0	3
P. tolaasii 6264		2	0	0	0	0	0	0	0	10	1	0	797	151	0	0	0	20	0	0	0	0	0
P. tolaasii PMS117		4	0	0	0	0	0	0	0	10	8	0	824	143	1	0	0	33	0	0	0	0	0
P. fluorescens BRIP34879		12	62	0	0	0	0	0	0	21	0	0	144	21	0	0	0	0	4	0	0	0	0
P. extremaustralis 14-3		20	168	4	1	0	0	0	0	4	0	0	0	2	0	0	0	3	201	0	0	0	0
P. fluorescens BS2		55	181	0	0	0	0	0	0	10	0	0	1	0	0	0	0	1	0	0	0	0	0
P. fluorescens WH6		9	41	0	0	0	0	1	2	4	0	0	2	0	0	0	0	27	0	0	0	0	17
P. sp. UK4		0	0	0	0	0	0	0	0	0	0	0	0	0	0	0	0	0	2	0	0	0	0
P. psychrophila HA-4	E	0	0	0	0	0	0	0	1	2	0	0	0	4	1	0	0	0	21	0	9	0	0
P. fragi A22		0	0	0	0	0	0	0	0	0	0	0	1	17	116	0	0	0	155	0	0	0	0
P. fragi B25		0	0	0	0	0	0	0	2	17	4	0	67	163	2	0	0	0	0	0	0	0	0
***P. fluorescens* Pf0-1**	F	0	0	0	0	0	0	0	3	10	10	0	0	14	31	7	0	0	0	0	0	0	0
P. sp. GM25		0	0	0	0	0	0	0	29	27	66	0	0	4	9	24	8	0	0	0	0	0	0
P. sp. R62		0	0	0	0	150	0	0	0	5	832	7	0	43	145	0	2	0	6	0	99	0	0
P. sp. GM30		0	0	0	0	139	0	0	3	51	582	249	0	19	178	7	0	380	13	0	97	0	0
P. fluorescens R124		0	0	0	0	37	0	0	1	1009	265	217	0	1	30	5	2	261	12	0	59	0	0
P. fluorescens NZ011		0	0	0	0	0	0	0	2	2	1035	240	0	0	6	4	2	2	12	0	111	0	0
P. sp. GM16		0	0	0	0	0	0	0	0	0	159	272	1	14	357	2	8	2	9	0	323	0	0
P. sp. GM24		0	0	0	0	0	0	0	0	0	153	261	1	14	325	2	8	2	9	0	304	0	0
P. sp. GM80		0	0	0	0	306	0	0	0	1	9	182	0	88	535	2	0	4	11	2	0	0	0
***P. sp.* UW4**	G	0	0	0	0	0	0	84	427	363	435	0	14	60	48	0	0	2	398	0	25	0	0
P. sp. GM33		0	0	0	1	0	0	92	438	540	156	0	7	34	21	0	0	58	272	0	145	0	0
P. sp. GM48		0	0	1	1	0	0	64	48	1283	8	0	1	5	0	0	0	212	233	0	1	0	0
P. sp. GM49		0	0	1	0	0	0	108	550	151	47	1	4	79	3	0	0	3	502	0	2	0	0
P. sp. GM55		0	0	391	90	0	0	1	33	15	45	0	4	203	35	0	0	1	435	0	0	1	0
P. sp. GM74		0	0	1	0	0	0	0	232	428	11	0	2	120	55	0	0	19	50	0	19	0	0
P. sp. GM78		0	0	17	0	0	0	0	9	12	101	92	1	188	114	117	7	0	150	12	70	53	0
P. fluorescens NCIMB 11764	H	0	0	2	2	51	0	1	2	13	11	0	1	11	5	59	31	186	2	0	12	2	0
P. mandelii JR-1		0	0	0	1	2	0	2	6	5	166	84	0	5	2	3	8	164	68	0	0	355	0
P. fluorescens HK44		0	0	0	0	0	0	56	10	5	29	0	1	0	0	0	0	304	1	100	1	1	0
P. sp. GM50		0	0	0	0	3	0	392	1	198	32	0	4	20	0	15	11	13	4	0	67	667	0
P. sp. GM102		0	0	0	0	0	0	140	2	77	6	0	16	116	1	11	14	245	0	0	57	679	0
P. sp. GM79		0	0	1	400	0	0	16	2	1417	8	0	9	62	0	0	0	127	0	0	168	75	0
P. sp. GM60		0	0	31	398	0	0	0	1	175	30	50	0	5	3	17	6	0	297	0	1	379	0
P. sp. GM67		0	0	20	301	0	0	0	2	33	41	23	0	4	1	37	26	0	101	1	0	656	0
P. sp. GM21		0	0	3	0	1	0	0	12	48	147	20	4	35	4	38	9	0	11	0	0	0	0
P. sp. GM18		0	0	0	0	0	0	27	1	2	3	0	1	7	0	0	0	28	1	1	65	35	0
P. sp. GM41(2012)		0	0	0	1	55	0	73	7	72	91	13	2	13	0	19	5	0	5	174	89	24	0
P. fluorescens Q2-87	I	0	0	0	0	47	0	0	75	576	130	0	0	2	0	0	0	586	2	1	0	0	0
***P. fluorescens* F113**		0	0	0	0	331	0	0	749	60	91	0	0	0	0	0	0	54	3	198	9	0	0
P. fluorescens Q8r1-96		0	0	0	0	30	0	0	661	61	109	0	0	0	0	0	0	44	5	295	17	0	0
P. fluorescens Wood1R		0	0	0	0	21	0	0	290	26	62	0	0	1	0	0	0	37	6	181	15	0	0
***P. brassicacearum* NFM421**		0	0	0	0	23	0	0	632	60	116	0	0	1	0	0	0	46	6	303	14	0	0

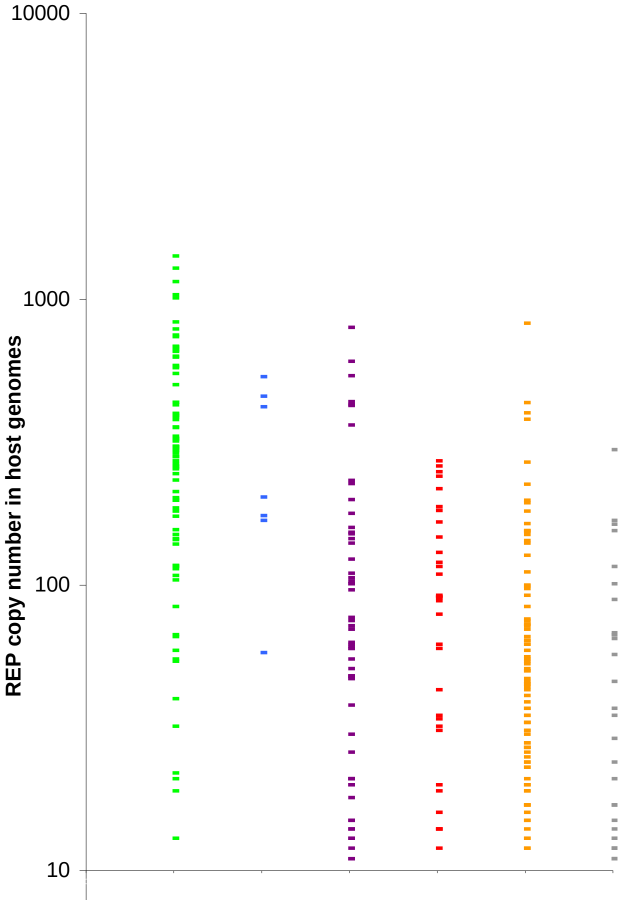

10000
1000
100
10
REP copy number in host genomes
