# Supplementary material for: Evolution of REP diversity: a comparative study
Source: BMC Genomics. 2013 Jun 10;14:385. doi: 10.1186/1471-2164-14-385 (PMC3686654; doi:10.1186/1471-2164-14-385)
Supplement: Additional file 4 — Sorting of REP copy numbers in stenotrophomonads based on their association with RAYTs. [file 1471-2164-14-385-S4.pdf]

Each field in the following table and each point in the following graph correspond to the copy number of particular REP class in host genome (cases of 10 or less REP occurrences per genome were discarded).

The colors mark one of the following conditions:

Green – intact cognate RAYT is present

Blue – pseudogenized cognate RAYT is present

Purple – cognate RAYT is absent, RAYT of the same orthogroup is present, cognate RAYT is present in strains of the same clade

Red - cognate RAYT is absent, RAYT of the same orthogroup is present, cognate RAYT is absent in strains of the same clade

Orange - cognate RAYT is absent, RAYT of the same orthogroup is absent, cognate RAYT is present in strains of the same clade

Grey - cognate RAYT is absent, RAYT of the same orthogroup is absent, cognate RAYT is absent in strains of the same clade

Bacterial strain	Clade	REP copy number												
		Ortho group I			Ortho group II		Ortho group III		NO*	NO*	NO*	NO*	NO*	NO*
		SM 1	SM 2	SM 3	SM 4	SM 5	SM 6	SM 7	SM 8	SM 9	SM 10	SM 11	SM 12	SM 13
S. maltophilia PML168	A	1	0	0	5	**18**	0	0	37	0	2	1	0	**96**
S. maltophilia S028		0	0	0	0	0	0	**45**	3	0	4	0	0	4
***S. maltophilia* R551-3**	no	**39**	4	16	62	1	6	0	266	**49**	**259**	49	18	0
S. sp. SKA-14	no	7	**37**	12	**128**	1	0	0	**323**	3	7	31	**82**	2
***S. maltophilia* D457**	B	31	8	**18**	37	2	3	0	**258**	**92**	5	15	0	2
***S. maltophilia* JV3**		**57**	4	7	**183**	2	1	0	**283**	9	10	**108**	1	1
S. maltophilia RR-10	C	18	**8**	10	98	0	6	0	47	**106**	**120**	15	2	0
P. geniculata N1		***33***	7	10	99	1	**12**	0	61	**116**	**107**	18	1	0
***S. maltophilia* K279a**		**52**	16	11	105	2	33	0	**427**	7	13	3	2	1
S. maltophilia Ab55555		**55**	13	9	***102***	4	**31**	0	**375**	6	12	3	1	1

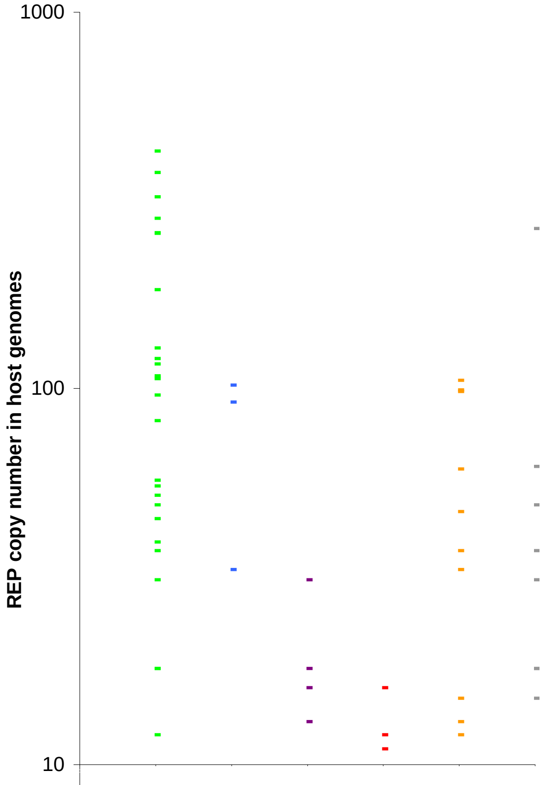

1000
100
10
REP copy number in host genomes
